# Tablet or capsule form of generic mycophenolate mofetil (My-Rept^®^) after liver transplantation: a prospective randomized trial

**DOI:** 10.2147/DDDT.S204056

**Published:** 2019-07-02

**Authors:** Jong Man Kim, Jong Wook Oh, Sangjin Kim, Jinsoo Rhu, Ji Soo Lee, Kyeong Sik Kim, Gyu-Seong Choi, Jae-Won Joh

**Affiliations:** 1 Department of Surgery, Samsung Medical Center, Sungkyunkwan University School of Medicine, Seoul, Republic of Korea

**Keywords:** tacrolimus, liver transplantation, efficacy, immunosuppression

## Abstract

**Background:**

Tablet and capsule forms have advantages and disadvantages in the market. Generally, the tablet form (500 mg) of mycophenolate mofetil (MMF) is more convenient for drug ingestion and more cost-effective than the capsule form (250 mg). We examined the efficacy and safety of MMF in its different forms combined with tacrolimus in liver transplant recipients.

**Methods:**

A randomized controlled trial was performed to compare the efficacy and safety between the tablet form of MMF (tablet group) and the capsule form of MMF (capsule group) in liver transplant patients. One hundred sixteen patients were enrolled in the present study from 2014 to 2017. Fifty-six patients in the full-analysis set (FAS) population were in the capsule group and 60 were in the tablet group. The primary endpoint was incidence of biopsy-proven acute rejection (BPAR) by 24 weeks after liver transplantation (LT). Secondary endpoints were patient survival, serum creatinine level, and adverse events (AEs).

**Results:**

In the per-protocol population, 45 patients were in the tablet group and 49 were in the capsule group. There were no statistically significant differences in MMF dose, mycophenolic acid trough level, and tacrolimus trough level between the two groups. The incidence of BPAR at 24 weeks after randomization was 6.7% in the tablet group and 6.1% in the capsule group (*P*=0.627). All patients with BPAR responded well to steroid pulse therapy and increased tacrolimus. Serum creatine level and eGFR were not different between the two groups. The incidence of serious AEs was 7.2% in the tablet group and 7.6% in the capsule group, and none were related to formulation. There was no significant difference in incidence of discontinuations or serious AEs between the two groups.

**Conclusion:**

The present study suggests that the new tablet formulation can be a useful treatment option to maintain a consistent systemic exposure level of MMF, which may help reduce graft failure in liver transplant patients.

## Introduction

Mycophenolate mofetil (MMF) is the most common immunosuppressant used to relieve calcineurin inhibitor (CNI)-related complications because of its low toxicity. In addition, MMF has a CNI-sparing effect with the ability to reduce acute rejection or graft failure.^1^,^2^ However, MMF is associated with leukopenia and gastrointestinal (GI) complications such as vomiting, diarrhea, and abdominal pain. These often require reduction of MMF dose.^1^,^3^

Cellcept (Roche Pharmaceuticals Corporation, Basel, Switzerland) is the original formulation of MMF. My-Rept (Chong Kun Dang Pharmaceutical Corporation, Seoul, Korea) is a generic form of MMF that was approved by the Korean Ministry of Food and Drug Administration (KFDA) in 2008 (for capsule form) and 2010 (for tablet form). The pharmacokinetic profile of an MMF generic formulation (My-Rept tablet and My-Rept capsule) is equivalent to those of Cellcept.^3^,^4^

The My-Rept 500 mg tablet form was considered bioequivalent to the Cellcept 500 mg capsule form according to the KFDA rulings, but no clinical studies regarding the efficacy and safety of tablet formulation usage have been conducted in LT patients receiving My-Rept. Therefore, we compared the efficacy and safety of the tablet formulation and the capsule formation of MMF in patients who had undergone LT.

## Materials and methods

### Study design

This study was a prospective, randomized, single-center, clinical trial in recipients (age ≥20 years) of a first LT from living or deceased donor. Inclusion criteria were primary liver transplantation; 20–65 years of recipients; and voluntary consent. Exclusion criteria were multiorgan recipients or previous transplant of any organ; liver donated after cardiac death; leukopenia (<1,500/mm^3^) and/or serum creatinine >2.0 mg/dL prior to enrollment; use of any other investigation drug within 4 weeks before screening; malignancy within the last 5years except squamous cell carcinoma or basal cell carcinoma in skin or primary hepatocellular carcinoma (HCC); donor with positive HBsAg; positive HIV status in donor or recipient; history of liver support system; evidence of HCC portal vein tumor thrombosis in preoperative radiologic images; presence of severe GI complications such as diarrhea or severe peptic ulcer disease at screening; women of childbearing potential who were unwilling to use an effective form of contraception for the duration of the study; women who were pregnant or lactating; and people who could not communicate because of psychological problems.

Written informed consent was obtained from all patients following institutional review board approval (SMC-2013-10-033), and the study was conducted in accordance with the guidelines for Good Clinical Practice, applicable local regulations and the Declaration of Helsinki.

### Immunosuppression

The patients who were randomized received basiliximab (Simulect, Novartis,Basel, Switzerland) induction, immediate-release tacrolimus (Tacrobell, Chong Kun Dang Pharmaceutical Corp.), MMF (My-Rept from Chong Kun Dang, Pharmaceutical Corp.) in either capsule or tablet form, and corticosteroids. Basiliximab was given just prior to transplantation and 4 days after transplantation. Dosage was maintained at a tacrolimus trough level of 3–8 ng/mL for both groups throughout the study period. Methyl-prednisolone tapered to a maintenance oral dose of >4 mg a day and discontinued within 3 months after LT.

My-Rept tablets (500 mg) and My-Rept capsules (250 mg) were administered to the tablet and capsule groups, respectively. The target dose of MMF was 500 mg twice daily (b.i.d.) for both groups at the beginning of the study. The maximum recommended dose was 2,000 mg, and recipients with more than two discontinuation episodes of MMF or who discontinued for >14 days were excluded. Both groups received MMF within 24 hrs after successful liver transplantation.

### Assessment

Study visits took place within 4 weeks before transplantation (screening); on the transplantation day; and at 4 weeks, 8 weeks, 12 weeks, and 24 weeks post-transplant. At each visit, a complete physical examination was performed, and laboratory values concerning the kidney, liver, hematology were calculated. Monitoring of MPA trough concentration was performed routinely alongside with tacrolimus trough concentration. Both whole blood tacrolimus trough concentration and plasma MPA trough concentration were monitored by high-performance liquid chromatography with tandem mass spectrometry.^5^,^6^ Blood pressure, weight, and any problems between visits were documented. We examined the renal function with serum creatinine level and with eGFR by the Modification of Diet in Renal Disease (MDRD) formula.^7^ Data were recorded, entered into an electronic database, and re-evaluated by external monitors. Study monitoring and database analyses were performed, and all adverse events (AEs) and serious adverse events (SAEs) were documented.

### Endpoints

The primary efficacy endpoint was biopsy-proven acute rejection (BPAR) and the secondary endpoints were graft failure and patient death by 24 weeks after transplantation. Patients with clinical findings suggestive of acute rejection underwent biopsy, and biopsy specimens were graded according to Banff criteria.^8^

### Statistical analysis

A sample size of 58 for each treatment group was determined for the primary endpoint by assuming a significance level (α) of 0.025, 80% power, 15% noninferiority margin, and a 15% dropout rate. Eligible individuals were randomly assigned in a 1:1 ratio to either the investigational or control group, the tablet group or the capsule group, respectively. Randomization assignments were centrally released via an electronic case report form prior to transplantation. For randomization of enrolled subjects, a random seed with a stratification factor for each research institution was generated. Block and block size were randomly assigned.

Categorical variables were analyzed using Chi-square test or Fisher’s exact test with SPSS software version 22.0 (SPSS Inc., Chicago, IL, USA). All categorical values were expressed as a percentage of the group. Continuous variables were analyzed using Mann–Whitney U test and expressed as median and range. In this study, *p*-values <0.05 were considered significant.

## Results

### Baseline characteristics

**Figure 1:**
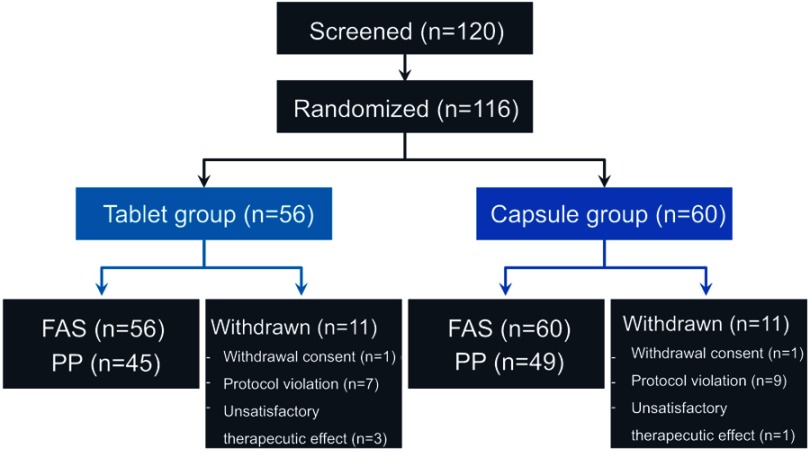
Patient distribution and study population.

Patients were screened from February 2014 to March 2017. Of 120 screened patients, 116 (56 in the tablet group and 60 in the capsule group) were randomized after transplantation and comprised the full-analysis set (FAS) population. Eleven patients in each group were excluded because of protocol violations, withdrawn consent, or unsatisfactory therapeutic effects. A total of 94 (45 in the tablet group and 49 in the capsule group) patients completed the study drug regimen and follow-up; they comprised the per-protocol (PP) population (Figure 1). There were no statistically significant differences in donors and recipients between the two groups (Table 1).

**Table 1:** Baseline characteristics

	Tablet group (n=56)	Capsule group (n=60)	*P*-value
**Donors**			
Gender (male)	32 (57.1%)	38 (64.4%)	0.425
Age (years)	33 (16–79)	32 (17–73)	0.631
Relationship (LDLT)	43 (76.8%)	45 (76.3%)	0.885
**Recipients**			
Gender (male)	43 (76.8%)	42 (70.0%)	0.409
Age (years)	57 (34–66)	55 (36–70)	0.132
Body mass index	24.2 (18.7–34.2)	24.3 (16.9–34.7)	0.599
ABO incompatibility	9 (16.1%)	21 (35.0%)	0.033
Diagnosis (HBV)	33 (58.9%)	39 (65.0%)	0.315
Coexistence of HCC	32 (57.1%)	38 (63.3%)	0.312
MELD score	14 (6–40)	15 (6–40)	0.835

**Abbreviations:** LDLT, living donor liver transplantation; HBV, hepatitis B virus; HCC, hepatocellular carcinoma; MELD, model for end-stage liver disorder.

### Tacrolimus trough level

**Figure 2:**
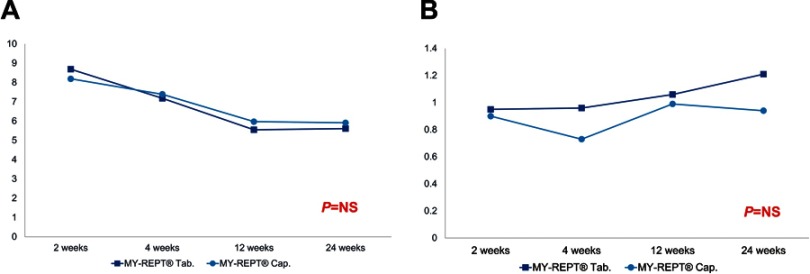
Blood trough levels of tacrolimus (**A**) and MPA (**B**) in the PP population.

The mean blood trough level of tacrolimus at 2, 4, 12, and 24 weeks post-transplant is shown in Figure 2(A). The mean blood trough levels of tacrolimus were not significantly different between the two groups for each study period (Table 2).

**Table 2:** Trough levels of tacrolimus and mycophenolate acid after liver transplantation (mean ± SD)

	Tablet group	Capsule group	*P*-value
Tacrolimus			
2 weeks	8.69±3.06	8.19±3.21	0.393
4 weeks	7.18±2.66	7.39±3.72	0.848
12 weeks	5.55±3.78	5.97±2.91	0.210
24 weeks	5.61±2.82	5.91±2.50	0.423
Mycophenolic acid			
2 weeks	0.95±0.74	0.90±1.15	0.337
4 weeks	0.96±0.97	0.73±0.80	0.202
12 weeks	1.06±1.28	0.99±1.32	0.822
24 weeks	1.21±1.16	0.94±0.80	0.275

### Mycophenolate acid (MPA) level and dose of MMF

In the PP population, the mean plasma level of MPA at 2, 4, 12, and 24 weeks post-transplant is shown in Figure 2(B). The mean MPA levels were not significantly different between the two groups for each study period (Table 2). The doses of MMF at the same times in the FAS population are outlined in Table 3. The proportions of patients receiving 1,000 mg/day in the tablet and capsule groups were higher than those in any other dosage groups at 0, 2, 4, and 12 weeks post-transplant. However, the proportions of patients receiving 500 mg/day in the tablet and capsule groups were higher than those in any other dosage groups at 24 weeks post-transplant.

**Table 3:** Optimum dose of MMF

Tablet group (mg/day)	Initial dose (n=56)	2 weeks (n=55)	4 weeks (n=53)	12 weeks (n=53)	24 weeks (n=48)
None	0	0	2	2	1
500	1	5	8	17	33
1,000	54	49	43	34	14
1,500	1	1	0	0	0
Capsule group (mg/day)	Initial dose (n=60)	2 weeks (n=60)	4 weeks (n=59)	12 weeks (n=57)	24 weeks (n=52)
None	0	3	1	1	2
250	0	0	3	0	0
500	5	7	18	19	33
750	0	0	0	1	2
1,000	51	48	35	33	13
1,500	4	2	2	3	2

### Efficacy

In the PP population, the overall incidence of BPAR in the tablet group was 6.7% (3/45) compared to 6.1% (3/49) in the capsule group (*P*=0.627). Among the patients with BPAR, two patients in the tablet group received steroid pulse therapy and one patient received increased immunosuppression, while one patient in the capsule group received steroid pulse therapy and one patient received increased immunosuppression. In addition, one patient in the capsule group was observed only. All patients with BPAR had good response to management and recovered to normal liver function. No graft loss or no patient death was reported in either group (Table 4).

**Table 4:** Efficacy in the PP population

	Tablet group (n=45)	Capsule group (n=46)	*P*-value
Biopsy-proven acute rejection	3 (6.7%)	3 (6.1%)	0.627
Graft failure	0 (0%)	0 (0%)	1.000
Death	0 (0%)	0 (0%)	1.000

In the FAS population, median white cell count, platelet count, aspartate transaminase, alanine transaminase, total bilirubin, and albumin at 2, 4, 12, and 24 weeks post-transplant were not different after LT (data not shown). In addition, renal function measures of median serum creatinine and eGFR in the tablet group were not different from those in the capsule group (data not shown).

### Safety

**Figure 3:**
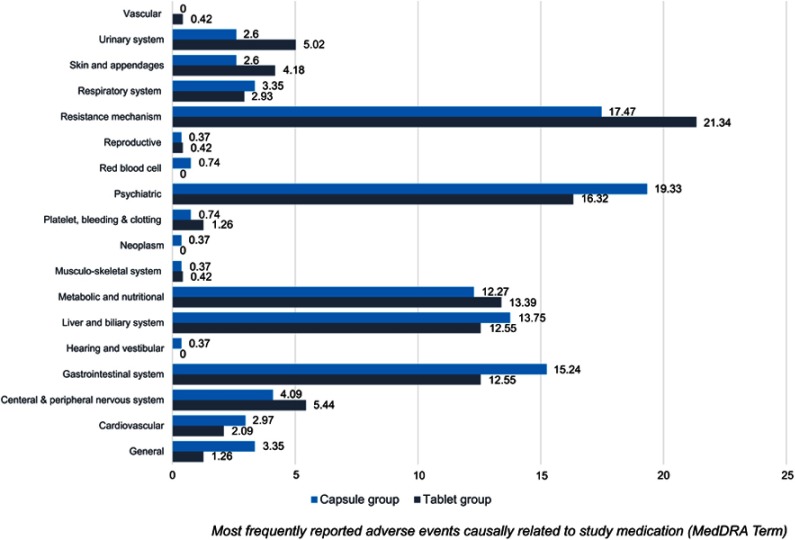
Adverse event profiles were similar between the tablet group and the capsular group.

The incidence of AEs is summarized in Table 5 and was similar between the groups. Among 116 patients in the FAS population, 21 patients (37.5%) in the tablet group and 19 patients (31.7%) in the capsule group reported an SAE during the study period. The incidences of AEs and SAEs were not significantly different between the groups (*P*=0.238 and *P*=0.320, respectively). Seventeen cases (7.2%) in the tablet group and 25 cases (7.6%) in the capsule group reported an SAE during the study period (*P*=0.248). The incidence of AEs by system organ class was generally similar between the groups (Figure 3).

**Table 5:** Summary of adverse events in the FAS population

	Tablet group (n=56)	Capsule group (n=60)	*P*-value
Number of patients with any AEs	48 (85.7%)	56 (93.3%)	0.238
Number of patients with SAEs	21 (37.5%)	19 (31.7%)	0.320
Adverse event cases	237	264	NA
Serious AEs cases	17 (7.2%)	25 (7.6%)	0.248

**Abbreviations:** AEs, adverse events; SAEs, serious adverse events; NA, non applicable.

## Discussion

Generic MMF (My-Rept), a 250-mg capsule produced by Chong Kun Dang Pharmaceutical Corporation, was first introduced and approved by the KFDA in 2008. After comparing the pharmacokinetic profiles of the Cellcept 250-mg capsule and the My-Rept 250-mg capsule, the KFDA stated that the two products were bioequivalent according to the KFDA-assigned range.^4^ Clinical experience and research data also demonstrated that the two products were comparable in terms of efficacy, AEs, and acceptable safety findings.^3^ The usual forms of brand-name MMF (Cellcept) and generic MMF (My-Rept) are 250-mg capsules.

Patients may have a harder time swallowing a capsule than a tablet because the floating property of the capsule makes it lighter than water, causing an uneasy globus sensation. In contrast, tablets are typically heavier than water, which could minimize the uneasy feeling in the oral cavity when swallowing.^9^ Thus, the tablet formulation may help alleviate patient discomfort, leading to increased compliance, better quality of life, and possibly better efficacy. Tablets undergo a more efficient and scalable manufacturing process than that used for capsule manufacture and are considered a preferred pharmaceutical dosing formulation for higher unit volume commercial production. A 500-mg tablet form of generic MMF (My-Rept), produced by Chong Kun Dang Pharmaceutical Corporation, was approved by the KFDA in 2010.^4^ Therefore, a bioequivalence investigation should be performed to characterize exposure to the tablet formulation relative to the current marketed capsule formulation.

In this prospective, randomized study, we investigated the efficacy and safety of different forms (tablet or capsule) of generic MMF in LT recipients. The present study showed a similar incidence of BPAR: 6.7% in the tablet group vs 6.1% in the capsule group. No patients in the PP population developed graft failure or died. The incidence of efficacy failure was not significantly different according to the form of MMF. In addition, the new tablet form of MMF with basiliximab induction, tacrolimus, and corticosteroids appeared to be efficacious for preventing acute rejection or graft failure.

MMF has been widely used to improve the renal function commonly associated with CNI.^1^ The dose of MMF and trough level of MPA in both groups were similar at post-transplant. We tapered the MMF dose of 500 mg/day at the post-transplant 24 weeks because of the possibility of rejection and/or AEs. Serum creatinine and eGFR levels were not different between the two groups at regular visits. Therefore, the tablet formation is not likely to affect renal function. Data on the optimal dose of MMF in combination with tacrolimus are scarce. Moreover, no studies have compared the different forms of MMF in LT recipients, especially in the Asian population. The costs of MMF in the commercial market in Korea are as follows: Cellcept 250-mg capsules, US $0.8; My-Rept 250-mg capsules, US $0.7; and My-Rept 500-mg tablets, US $1.1 (all values were converted using the 2018 exchange rate). The costs for a 1,000 mg dose according to dosage form are as follows: Cellcept 250-mg capsules, US $3.2; My-rept 250-mg capsules, US $2.9; and My-rept 500-mg tablets, US $2.2. When we consider MMF usage during the long-term period, My-Rept^®^ tablet is cheaper than My-Rept^®^ capsule or Cellcept^®^. Therefore, the present study suggests that the tablet form of MMF (500 mg) may be more convenient in terms of drug ingestion and more cost-effective than the capsule form (250 mg) for LT recipients. The rates of reported SAE were similar (7.2% in the tablet group vs 7.6% in the capsule group). The formulations appeared to be equally well tolerated by liver transplantation recipients. SAEs were not treatment-related drugs. In addition, no clinically relevant differences in overall safety profiles of the two formulations were evident.

In conclusion, liver function tests, renal function, and incidence of BPAR in the tablet group at 24 weeks post-transplant were not inferior to those of the capsule group. The present study suggests that the new tablet formulation can be a useful treatment option to maintain a consistent systemic exposure level of MMF, which may help reduce graft failure in liver transplant patients. In addition, these beneficial characteristics of the tablet formulation of MMF make it a useful alternative to the conventional capsule formulation after LT. The bioequivalent 500-mg My-Rept tablet is efficient, safe, cost-effective, and convenient for patients after LT. However, the present study focused on the short-term outcome of tablet form, and further studies should be needed on the long-term effect of the tablet in terms of economic factors or adverse reactions.
